# TDP‐43 dysfunction and markers in Neurodegenerative Disease

**DOI:** 10.1002/alz70855_104405

**Published:** 2025-12-24

**Authors:** Leonard Petrucelli

**Affiliations:** ^1^ Mayo Clinic, Jacksonville, FL, USA

## Abstract

**Background:**

Although Alzheimer's disease (AD) is classically defined by the accumulation of tau neurofibrillary tangles and Aβ plaques, a large proportion of cases are also positive for inclusions containing TAR DNA binding protein of 43 kDa (TDP‐43) – and evidence shows that these cases generally have a more aggressive disease course marked by worse memory and increased hippocampal atrophy. As we advance towards precision medicine in AD, it is imperative that we understand the contribution of TDP‐43 proteinopathy to pathogenesis and develop tools to track this pathology in living patients.

**Methods:**

In affected neurons, TDP‐43 nuclear clearance triggers mRNA misprocessing events including cryptic exon inclusion and alternative polyadenylation. We aim to exploit these events to develop sensitive biomarkers that can serve as proxy measures for TDP‐43 dysfunction. As we identify misprocessed TDP‐43 targets that accumulate in affected brain regions in AD, we also explore the impact of these aberrant transcripts on molecular processes and pathways in the brain to gain insight into disease pathomechanisms.

**Results:**

We have developed tools to measure a promising biomarker of TDP‐43 dysfunction, a cryptic form of the protein HDGFL2, and verified that it can be detected in post‐mortem AD patient tissue specifically in brain regions marked by mixed pathology. We have also uncovered a connection between TDP‐43 dysfunction and compromised endolysosomal activity. In fact, we find that artificially severing the endolysosomal pathway via expression of a constitutively active Rab5 can induce TDP‐43 pathology and neurodegeneration *in vivo* in mice, and we are using this tool to map the consequences of endolysosomal dysfunction in the brain and its potential connection to mixed pathology formation in AD.

**Conclusions:**

TDP‐43 co‐pathology has a profound effect on AD pathogenesis that may be intrinsically linked to a disruption of the endolysosomal pathway in neurons. Exploring the consequences of TDP‐43 dysfunction in the brain can open new avenues for AD biomarker and therapeutic development.

